# Robot Navigation Based on Potential Field and Gradient Obtained by Bilinear Interpolation and a Grid-Based Search

**DOI:** 10.3390/s22093295

**Published:** 2022-04-25

**Authors:** Gregor Klančar, Andrej Zdešar, Mohan Krishnan

**Affiliations:** 1 Faculty of Electrical Engineering, University of Ljubljana, Tržaška 25, 1000 Ljubljana, Slovenia; gregor.klancar@fe.uni-lj.si (G.K.); andrej.zdesar@fe.uni-lj.si (A.Z.); 2 Electrical & Computer Engineering and Computer Science Department, University of Detroit Mercy, Detroit, MI 48208, USA

**Keywords:** robot navigation, path planning, potential field, bilinear interpolation, dynamic local re-planning

## Abstract

The original concept of the artificial potential field in robot path planning has spawned a variety of extensions to address its main weakness, namely the formation of local minima in which the robot may be trapped. In this paper, a smooth navigation function combining the Dijkstra-based discrete static potential field evaluation with bilinear interpolation is proposed. The necessary modifications of the bilinear interpolation method are developed to make it applicable to the path-planning application. The effect is that the strategy makes it possible to solve the problem of the local minima, to generate smooth paths with moderate computational complexity, and at the same time, to largely preserve the product of the computationally intensive static plan. To cope with detected changes in the environment, a simple planning strategy is applied, bypassing the static plan with the solution of the A* algorithm to cope with dynamic discoveries. Results from several test environments are presented to illustrate the advantages of the developed navigation model.

## 1. Introduction

The main goal of a navigation function is to create feasible, safe paths that avoid obstacles and allow a robot to move from its start configuration to its goal configuration [1]. Online robot navigation and path planning consists of two complementary aspects. In the global path-planning phase, the task is to find an optimal path to the intended goal, starting from the robot’s starting position and using all the previous information about the environment. This plan takes into account the need to avoid obstacles but only those that are assumed to be present before the robot starts to move towards the goal. This is coupled with the local obstacle avoidance phase in which the robot avoids new obstacles detected by its sensors while navigating the planned path. The former can be thought of as proactive, while the latter is reactive. With this understanding comes the acceptance that the former is usually more optimal than the latter in some sense. The biggest challenge in real-world applications is the ability to handle unanticipated changes in both structured and unstructured environments. Because the discovery of new obstacles is an evolutionary process, it cannot be assumed that the overall path that is eventually completed will be as optimal as a path that was planned with the knowledge of all the aspects of an unchanged environment at the beginning. However, this is not a fair comparison because new map situations are usually discovered after the robot has begun to execute the original plan. Moreover, due to these new discoveries, the robot may find itself in situations where it seems to be trapped if it continues to follow the global plan while only imposing a requirement to avoid collisions with the new obstacles. The goal is then to develop strategies to overcome the new obstacles in an effective and situationally appropriate way while the robot continues to head towards its original goal.

It is important to understand the trade-off involved in the above situation, which can be thought of as a see-and-react strategy when dealing with new discoveries. In contrast to this approach, there is the alternative of re-implementing the global path-planning strategy at the point where new environmental discoveries are made. However, implementing a full-featured online global path-planning strategy is usually not feasible due to computational costs. Speed is essential because potential delays in reacting could affect the ability to deal with new discoveries safely and efficiently unless one is willing to slow down or stop until a new plan is available. This is particularly true when considering that changes in the environment relative to prior assumptions are quite likely in actual navigation applications. It is to address this need that several incremental methods have been developed [2,3,4,5,6], which reduce computational and storage costs by reusing existing planning information.

An extensive survey of path planning algorithms has been carried out in [7]. Algorithms are divided into categories and sub-categories within them, based on the modality of their development. This work falls in the sub-category of graph search, which supports a variety of path-planning approaches, but here specifically, a graph search based on the use of a uniform grid. It combines this with the adoption of a variant of the original artificial potential fields (APF) method [8]. According to [7], the APF is categorised as reactive manoeuvring. Thus, essentially, a uniform grid-based graph search is combined with a reactive manoeuvring technique to carry out global and local path planning.

The environment can be represented as a graph using cell decomposition or roadmaps. Examples of the latter approach are the Voronoi graph [9], which can produce optimum clearance from the obstacles, and the tangent graph [10], which contains the optimal solution and requires less memory than the visibility graph, its superset. In [11], a tangent graph is constructed for obstacles described with analytic curves in which a finite search algorithm can be used to find the optimal path. The optimal path found in the tangent graph may not be smooth. The authors in [11] combine the tangent graph with online reactive navigation to generate smooth paths for the unicycle drive. Although the algorithm always finds a path, the path may not be optimal. The computational complexity of roadmap-based path-planning algorithms depends on the number of obstacles and the complexity of the obstacle shapes (i.e., the number of the primitives describing all obstacles). The problem of reducing the computational complexity of constructing a tangent graph was addressed in [12]. Among the cell decomposition approaches, grid-based tessellation is the most common and is also used in this work. In grid-based approaches, the complexity mainly depends on the number of cells—a smaller cell size leads to a higher path resolution. The cell size must be small enough to describe the environment with sufficient detail, but must not be too small, as this significantly increases the computational complexity of the search algorithms. The proposed approach introduces an interpolation that can produce smooth paths even at a coarse map resolution.

The earliest graph-theoretic path-planning algorithm is arguably the one developed by Dijkstra [13], which inspired many subsequent variations. It has two aspects associated with its basic construction that could make it less suitable for use in some real-world robot navigation problems. Firstly, it finds the optimal paths between a source node and all destination nodes (or equivalently, between multiple source nodes and a single destination node). In many applications involving only a single robot and a single destination, it increases the computational burden in doing much more than is needed. Secondly, it does not accommodate new discoveries made as the robot’s sensor horizon advances on the way to the goal. Yet, in other applications involving several missions originating at different locations, with the need to converge to a single destination such as in warehouses [14,15], or games [16,17], Dijkstra’s algorithm matches the need. The computational burden associated with it then becomes justifiable, especially if it is required to be exercised occasionally and the results reused with different starting locations. The focus then shifts to the second issue mentioned above, as to how to make the Dijkstra paths viable even when dealing with environmental changes.

The A* algorithm [18] and its derivatives, such as D*Lite [4], are computationally efficient algorithms compared to Dijkstra in the specific task that they address of finding a path between a single source and goal nodes. This reduced task allows an informed search strategy in the form of a heuristic to be deployed, which leads to the computational savings. The one-source–one-goal paradigm can be identified with a single robot trying to get to a single destination. Both A* and D*Lite accommodate new obstacle discoveries, but the latter is an incremental algorithm which makes it suitable when there are continuing map changes while navigating to the same goal.

Some of the prior efforts of other researchers that use methods related to our path planning and navigation strategy are now discussed. The concept of artificial or virtual potential functions (APF) was first proposed in [8]. They are called artificial because these are not actual electric potentials but are only conceptualised as such. In the original formulation, as explained in Choset [19], the two attractive and repulsive potential functions were algebraically summed to obtain the overall potential function. In practice, the ad hoc parametric choices of the model could set up local minima at which the net force on the robot is zero, resulting in the robot being trapped on its way to the goal.

Let us now turn our attention from the core APF concept to how it was actually realised in prior robot navigation research. Ratering and Gini [20] proposed a hybrid potential field consisting of the combination of a global potential field calculated with a variant of Dijkstra’s algorithm and a local potential field synthesised with the help of sonar measurements. Wang et al. [21] also constructed the global and local planners separately. Distance transformation, another variant of Dijkstra’s algorithm, is used for the global planner, while an APF-based method is used for the local planner. The overall navigation strategy is characterised by a mediation between the strict need to achieve the subgoals of the global plan and the freedom of the APF-based local planner, so that local minima are avoided. Azmi and Ito [22] propose a technique to handle the local minima problem, in this case, a repetitive oscillatory excursion between two local minima. A map transformation operation was proposed that resulted in the stalemate being resolved through the rotation of the environment space. Lazarowska [23] devised the planning of trajectories for autonomous ships navigating amongst both static and dynamic obstacles. The static APF model accounted for the compliance of special maritime rules that prescribed deliberate actions to avoid collisions between ships. Similarly, Klančar and Seder [15] combined the static APF with local reactive model–predictive planning to avoid collisions among multiple robotic vehicles in warehouse navigation. Amiryan and Jamzad [24] used the APF to complement a pre-determined path generated by a sampling-based path planner such as Rapidly-exploring Random Tree (RRT) [6] to avoid local minima problems. In [25], a hybrid planning method is proposed that combines a particle swarm optimisation algorithm with the APF for static obstacles and the potential field prediction for dynamic obstacles. Several solutions have been proposed to overcome problems with local minima, such as representing concave obstacles by convex representations [26], adding virtual obstacles to move away from local minima [27] or by small perturbations of the APF based on the input-to-state stability property [28]. Alternatively, a robot navigation function can be determined using deep neural networks with reinforced learning as in [29], ant colony optimisation [30], simulated annealing, particle swarm optimisation [25], genetic algorithms and fuzzy logic [31] or the like.

The sampling of the APF-based literature discussed above indicates that the APF concept continues to play a role in robot navigation. Specific use cases range from the original concept of an integrated formulation premised on attractive/repulsive forces to separate formulations addressing the static and dynamic planning phases, to dealing with dynamic moving obstacles and other variations.

The main contributions of this paper are the following.

- A new navigation function is proposed that generates smooth and collision-proof paths by using the bilinear interpolation (BiLI) method to obtain an artificial potential field gradient-descent navigation function from a discrete cost-to-goal (CtG) map obtained by an optimal discrete grid-based search method. The approach is computationally efficient as it relies on a coarse discrete graph search that can be precomputed for static environments and known goals and can be easily reused for multiple missions from different parts of the environment that need to navigate to a common goal. The bilinear interpolation method implements a continuous potential field and driving direction from a discrete grid-based search.
- Although BiLI is commonly used in computer vision applications, its use for robot planning requires some enhancements, such as handling occupied cells whose values are not defined, interpolating at the environmental boundaries and ensuring continuous gradient descent, which are the main novelties of this work. The resulting path is collision-proof, continuous and close to the optimum, even at the course grid resolution used. It also avoids the problems with local minima that are common in general APF-based methods.
- BiLI can be applied in dynamic environments where incremental graph search methods such as D${}^{*}$ or similar [2,4,5,32] can be used to efficiently account for the changes in the environment. In this work, a simple strategy is proposed using Dijkstra for the global CtG map planning and A${}^{*}$ for dealing with locally sensed environment changes. When different types of map changes are detected that affect the CtG values, the proposed model uses the A* algorithm to find an emergency bypass path to areas of the environment where the old CtG values are still valid. The bypass path is followed by the determination of a final gradient-descent path segment to the overall goal. Then, a situational decision is made whether to take the additional path segments at the point of the map change or to keep the original path. Using A* to determine the diversion path is relatively fast compared to regenerating the CtG values with Dijkstra, as usually only a few cells need to be examined.

The remaining parts of the article are organised as follows. The first part of the path-planning model developed in this effort is explained in Section 2 and Section 3, with the help of a test environment scenario. The reactive strategy to negotiate a blockage of the global path is formulated in Section 4 with the same environment. Section 5 contains a description of additional test scenarios formulated to evaluate this model and the attendant results. Finally, Section 6 contains a brief summarising discussion of the work and suggests further steps, while Section 7 draws some broad conclusions.

## 2. The Environment and APF Generation

Following a description of the environment, the formulation of the APF values for each cell of the grid-based discretisation is discussed in this section.

### 2.1. The Environment

**Figure 1:**
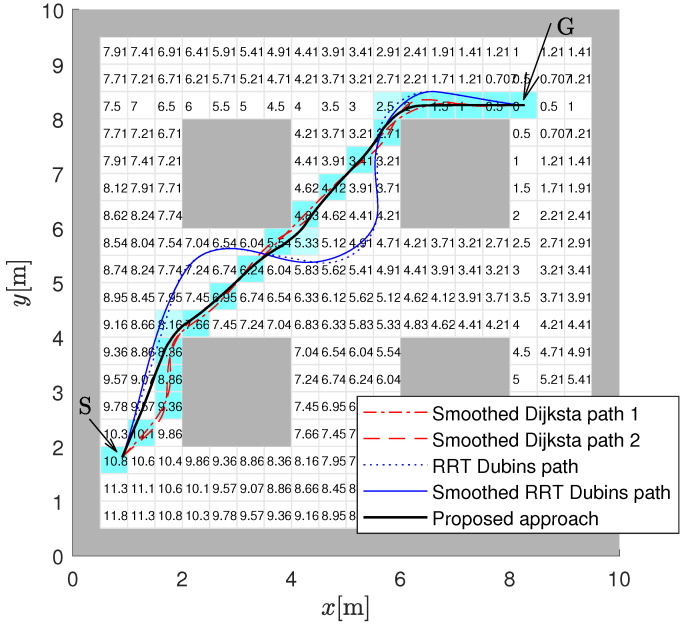
Static map of environment with start (S) and goal (G) locations, discrete CtG values assigned to the free cells (white cells, gray cells belong to obstacles) and some different smooth paths that connect the start and the goal locations.

A sample environment is shown in Figure 1 which represents the static map in one experiment. Appropriate modifications to it will be subsequently made that reflect the dynamic discovery of new obstacles. Moreover, other obstacle configurations will be created later that represent additional challenges addressed in this work.

The environment consists of a walled-off 10 by 10 m field of play. It can be scaled up to any size, as desired. The entire area is divided into 400 cells (20 by 20), with each cell being 0.5 m square. The environment features four prominent symmetrically positioned “obstacle islands” that, given the starting and goal locations (also shown), block line of sight to the goal for significant portions of a possible path.

The broad characteristics of the environment are:

- Even when an obstacle only overlaps part of a cell, the entire cell is considered occupied. Thus, the map of the environment is in the form of a binary occupancy grid, which also factors in obstacle inflation.
- Dynamic changes in the environment over the initial static knowledge are assumed to be small and localised.
- They can be in the form of additions or subtractions. That is, cells that were unoccupied might be occupied as well as the opposite.

### 2.2. Formulation of APF

A variant of the original APF (artificial or virtual potential functions) concept [8] is used to fulfill the global path-planning function. As pointed out earlier, the original APF was conceived as a function that is continuous with respect to space, that addressed both goal seeking and obstacle avoidance in an integrated manner. It followed from attractive and repulsive forces between artificial electrical charges. However, that approach is known to potentially spawn local minima trapping the robot.

As opposed to the classical approach, the floor space is tessellated into a suitable x–y grid to accommodate the use of a standardised discrete occupancy map to represent obstacles. The Dijkstra algorithm is used to generate the cost-to-goal (CtG) value for all cells in the environment, which constitutes the global cost map. In doing this, diagonal cell-to-cell transitions are given appropriate differential weights relative to horizontal and vertical transitions. The CtG values are shown overlaid in Figure 1 within each cell for the assumed environment. For example, the CtG is zero for the goal cell and 10.8 m for the starting cell.

Within the grid map, the Dijkstra algorithm can be used to obtain the shortest path. In Figure 1, the cells that lead from the start to the goal cell are shaded with a light blue colour. A smooth path can then be obtained if a spline (using, e.g., Bézier curves or clothoid curves) is fitted over the centres of the cells that comprise the path. In Figure 1, the Automated Driving Toolbox in Matlab is used to smooth the paths using cubic splines as shown in two examples (Smoothed Dijkstra path 1 and Smoothed Dijkstra path 2). This approach does not ensure that the smooth path does not go too close or even over the obstacles unless collision checking is also made during spline fitting. In Figure 1 is also an example of the path obtained by the approach proposed in this paper. This is a smooth path that is obtained based on the interpolated gradient of the APF that takes obstacles into account implicitly. Figure 1 also presents a smooth path that is obtained with Rapidly-exploring Random Tree (RRT*) [33]. In this case, RRT* uses Dubins’ curves [1] to obtain the path from the start to the goal, and the obtained path is further smoothed by fitting a cubic Bézier spline. The shape of the curve depends on many constraints (e.g., path curvature, segment length, safety distance, vehicle constraints, etc.) that can be given to the algorithm to optimise; therefore, paths with different shapes and smoothness can be obtained. Moreover, the RRT approach is stochastic; therefore, a completely different solution can be obtained in every run of the algorithm even if the input conditions do not vary. Some smoothed paths can be very oscillatory or make large turns around the obstacles, or path smoothing can also produce infeasible paths that collide with obstacles. The proposed approach in this paper is deterministic and produces smooth and near optimal paths that ensure a minimum safe distance from the obstacles, and it is also computationally efficient because it produces satisfactory results even when the cell size is relatively large.

The CtG numbers serve as the classical APF values that are the basis of the global planning within the environment, after some refinement discussed shortly. Just as in the classic APF method, a gradient descent determines the direction of motion. If the static map does not change, a gradient-descent approach based on the CtG values can be used to move the robot all the way to the goal. However, details need to be addressed, such as how the gradients are calculated and smoothed for a function that is discrete over the floor space, as well as how dynamic discoveries in the environment are handled, etc.

## 3. Interpolation and Smoothing of Potentials and Gradients for Path Planning

The discretisation of the floor—a decision made to contain the computational cost as well as to simplify the consideration of obstacle occupancy—correspondingly creates a discrete CtG surface. This then restricts the resolution of the gradient determination which affects the smooth navigation. To address this, a refinement is introduced through the use of a well-known technique of image resampling known as bilinear interpolation (BiLI) [34,35], which operates on pixels that are like the cells in our environment, as discussed below.

### 3.1. Bilinear Interpolation

**Figure 2:**
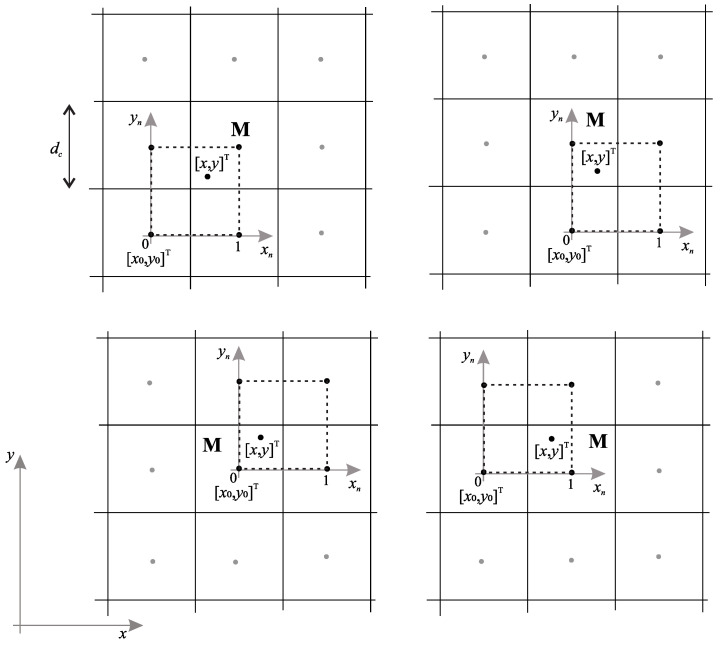
Basis of bilinear interpolation. Interpolated potential at a given point ${\left[x,y\right]}^{T}$ is defined by the discrete potential at centres (black dots) of four cells connected by the dashed square. Gray dots denote centres of cells.

The conceptual basis of BiLI is explained using Figure 2 [35]. BiLI uses a 2 by 2 cell window to interpolate CtG values within the centred unit square region within this window (dashed square in Figure 2), thus creating new data points in an educated manner. It does so by using linear functions to perform the interpolation in what is essentially a planar extension of 1D linear interpolation. The spline-based function representing the interpolating surface is associated with 4 parameters whose values need to be estimated. This is achieved using the CtG cell values at the corners of the unit square.

According to location of a point ${\left[x,y\right]}^{T}$ in a cell M, a 2 by 2 region of cells around it is chosen for the interpolation, whose centres are connected by a dashed square in Figure 2. Normalised coordinates are found by centres of these cells as:

(1) $$\begin{array}{ccc} x_{n}=\frac{x-x_{0}}{d_{c}} & , & y_{n}=\frac{y-y_{0}}{d_{c}} \end{array},$$

where $\left[x_{0},y_{0}\right]$ is the origin of the normalised coordinates defined by the lower left corner of the dashed square and $d_{c}$ is the cell size. The interpolated and discrete CtG value (potential in the sequel) in normalised coordinates are expressed as $P_{n}\left(x_{n},y_{n}\right)=P\left(x,y\right)$ and $U_{n}\left(x_{n},y_{n}\right)=U\left(x,y\right)$, respectively. The potential for the four adjacent cell centres (corners of dashed square in Figure 2) are

(2) $$p_{cr}=U_{n}\left(x_{n},y_{n}\right){|}_{x_{n}=c,\;y_{n}=r},$$

where c,r∈{0,1} and $U_{n}\left(x_{n},y_{n}\right)=U\left(x,y\right)$.

The interpolated potential $U_{n}\left(x_{n},y_{n}\right)$ at any given normalised position $\left[(x_{n},y_{n}\right){]}^{T}$ inside the quadrant of cell M delineated by the unit square is defined as [35]:

(3) $$P_{n}\left(x_{n},y_{n}\right)=w_{00}p_{00}+w_{01}p_{01}+w_{10}p_{10}+w_{11}p_{11},$$

where the BiLI weights are given by: $w_{00}=\left(1-x_{n}\right)\left(1-y_{n}\right)$, $w_{01}=\left(1-x_{n}\right)y_{n}$, $w_{10}=x_{n}\left(1-y_{n}\right)$ and $w_{11}=x_{n}y_{n}$.

By following the negative gradient of interpolated potential $P\left(x,y\right)=P_{n}\left(x_{n},y_{n}\right)$, the safe path from everywhere in the environment towards the goal location (with potential 0) can be obtained. The negative gradient of P(x,y) in ${\left[x,y\right]}^{T}$ can be obtained as:

(4) $$\begin{array}{ccc} g(x,y) & = & -\nabla P\left(x,y\right)=-{\left[\frac{\partial P(x,y)}{\partial x},\frac{\partial P(x,y)}{\partial y}\right]}^{T}-\frac{1}{d_{c}}{\left[\frac{\partial P_{n}\left(x_{n},y_{n}\right)}{\partial x_{n}},\frac{\partial P_{n}\left(x_{n},y_{n}\right)}{\partial y_{n}}\right]}^{T}\\ \; & = & -\frac{1}{d_{c}}\left[\begin{array}{c} p_{11}y_{n}-p_{01}y_{n}+p_{00}\left(y_{n}-1\right)-p_{10}\left(y_{n}-1\right)\\ p_{11}x_{n}-p_{10}x_{n}+p_{00}\left(x_{n}-1\right)-p_{01}\left(x_{n}-1\right) \end{array}\right]. \end{array}$$

### 3.2. Adjustments of Bilinear Interpolation for Path Planning

Before applying the interpolation of Equation (3), a check needs to be performed if any of the three neighbour cells of the cell M (see Figure 2) involved in the interpolation are occupied. Note that cell M is never occupied as we are interpolating potential at a point ${\left[x,y\right]}^{T}$ inside it. For occupied cells, the potential is typically infinite or undefined, as motion over the obstacles towards the goal should not be possible/permitted. The potential for occupied cell $U(x_{m},y_{m})$ (with centre at $x_{m}$, $y_{m}$) is reconstructed from the eight-cell neighbourhood by finding the unoccupied cell with the largest potential, as:

(5) $$\begin{array}{c} \left\{c,r\right\}=\underset{c,r}{\mathrm{argmax}}\left\{U(x_{m}+d_{c}c,y_{m}+d_{c}c)\neq \infty \right\}\\ U\left(x_{m},y_{m}\right)=U\left(x_{m}+d_{c}c,y_{m}+d_{c}r\right)+d_{c}\sqrt{c^{2}+r^{2}} \end{array},$$

where c,r∈{−1,0,1}.

Additionally, a check needs to be performed if any of the four cells needed for the interpolation (also interpolation cells, see Equation (3) and Figure 2) is outside the environment. A simple solution to this could be that the grid cell area is always at least one cell larger than the area we are interpolating. More general solution applies calculation of the potential and gradient for a nearby location ${\left[x_{t},y_{t}\right]}^{T}$, where all four cells used in the interpolation are inside the environment. For a position ${\left[x,y\right]}^{T}$, where one or more interpolation cells are outside the environment, the nearby location is determined by translating the position from the border for $\frac{d_{c}}{2}$ in *x* and/or *y* direction towards the inside of the environment as follows:

(6) $$x_{t}=\left\{\begin{array}{ccc} x & ; & x_{min}\leq x\leq x_{max}\\ x_{min}+\frac{dc}{2} & ; & x<x_{min}\\ x_{max}-\frac{dc}{2} & ; & x>x_{max} \end{array}\right.y_{t}=\left\{\begin{array}{ccc} y & ; & y_{min}\leq y\leq y_{max}\\ y_{min}+\frac{dc}{2} & ; & y<y_{min}\\ y_{max}-\frac{dc}{2} & ; & y>y_{max} \end{array}\right.,$$

where environment borders are defined by $x_{min}$, $x_{max}$, $y_{min}$, $y_{max}$. For translated nearby location, interpolated potential is computed from (3) noted as $P(x_{t},y_{t})$ and the gradient from (4) noted as $g_{t}\left(x_{t},y_{t}\right)$. Finally, the appropriate potential for each interpolating cell outside the environment (noted as $P^{*}$) are reconstructed using Lie derivative (also direction derivative):

(7) $$P^{*}=P\left(x_{t},y_{t}\right)+g_{t}\left(x_{t},y_{t}\right)\times {\left[x-x_{t},y-y_{t}\right]}^{T}.$$

**Figure 3:**
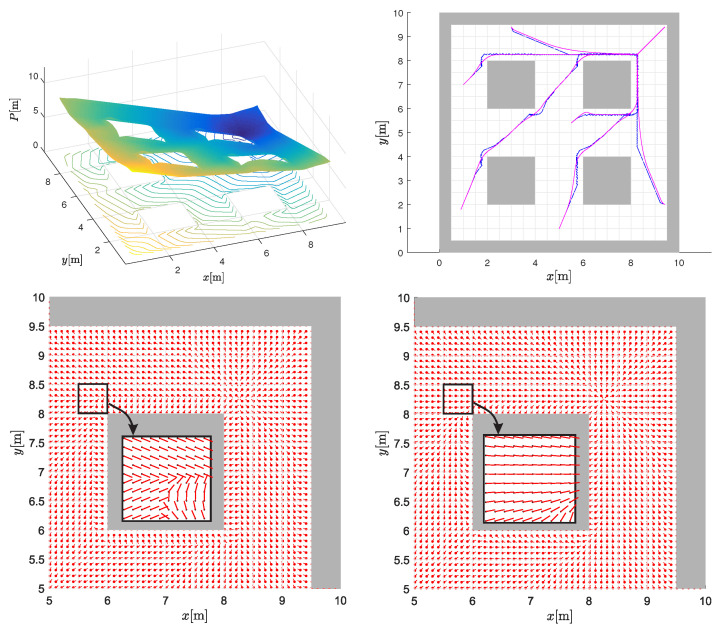
Interpolated potential surface $P_{n}\left(x_{n},y_{n}\right)$ (CtG values, darker colours denote lower CtG values) obtained through bilinear interpolation with contours of equal potential, corresponding to the discrete CtG values of environment of Figure 1 (**top-left**). Obtained paths following the negative gradient (blue line) and interpolated negative gradient (purple line) are shown (**top-right**). Part of the environment near the goal with negative gradients (red lines going from black dots outwards) computed from (4) (**bottom-left**) and interpolated negative gradients (**bottom-right**).

Figure 3 (top-left image) shows the resulting potential (CtG values) surface obtained through application of the BiLI technique, corresponding to the environment of Figure 1. Notice how the CtG surface slopes continuously downward from the start point to the goal point, with the four “islands” represented by infinite potentials. This follows from the fact that the CtG values will monotonically decrease from any cell in the environment towards the goal. Moreover, a few sample paths obtained by following the negative gradient (computed from Equation (4)) from different locations towards the goal are shown in the top-right image with blue line. Notice discontinuous change of the negative gradient direction near occupied cells, which can also be observed by checking gradients near obstacle (the cells near the central obstacle in the bottom-left image from Figure 3 with enlarged cell at obstacle corner). To improve this and obtain smoother paths (as illustrated in the top-right figure by purple line), we additionally propose interpolation of the negative gradients in Section 3.3.

### 3.3. Interpolation of Gradients

The resulting potential in Figure 3 is continuous, but its negative gradient may be discontinuous especially in the vicinity of obstacles, as seen from the bottom-left image in Figure 3 where, at the boundaries between the quadrants of a cell, the gradient shows discontinuities. The negative gradient indicates the required driving direction to reach the goal in optimal manner. Every discontinuity of the gradient is problematic, as a wheeled robot would need to stop and rotate on the spot to reliably follow the course of the negative gradient towards the goal. To improve this, we propose interpolation of the negative gradient, similar to what was performed for the potential field.

For chosen location ${\left[x,y\right]}^{T}$ in cell M, we estimated the interpolated potential P(x,y) and its negative gradient g using Equations (3) and (4). Interpolated potential is obtained from the four interpolation cells centre potentials as indicated by Figure 2. In the following, we will use the same interpolation principle to also obtain the interpolated negative gradient h(x,y) with continuous course as shown in the right images of Figure 3. The gradient for the centres of the four interpolating cells can be estimated from (4), which for each cell considers only the left neighbour ($x_{n}=0$) to obtain *x* element of the gradient g or only the upper neighbour ($y_{n}=0$) to obtain *y* element of g. Therefore, we estimate the cell centre gradient considering the smallest discrete potential (valid for the cell centre) of both neighbour cells in *x* or *y* axis. Denote the interpolating cell centre gradients (similarly as their potential in (2)) by $h_{cr}={\left[hx_{cr},hy_{cr}\right]}^{T}$ where c,r∈{0,1}. For a cell with centre coordinates $x_{m}=x_{0}+d_{c}c$, $y_{m}=y_{0}+d_{c}r$ the cell gradient reads

(8) $$h_{cr}=-\left\{\begin{array}{ccc} \frac{1}{d_{c}}\left[\begin{array}{c} e\left(U\left(x_{m}+d_{c}e,y_{m}\right)-p_{cr}\right)\\ f\left(U\left(x_{m},y_{m}+d_{c}e\right)-p_{cr}\right) \end{array}\right]\; & ; & U\left(x_{m},y_{m}\right)<\infty ,\;\\ \frac{1}{d_{c}}\left[\begin{array}{c} S_{x}\left(U\left(x_{m}+d_{c}S_{x},y_{m}\right)-p_{cr}^{*}\right)\\ S_{y}\left(U\left(x_{m},y_{m}+d_{c}S_{y}\right)-p_{cr}^{*}\right) \end{array}\right]\; & ; & U\left(x_{m},y_{m}\right)=\infty ,\; \end{array}\right.$$

where

(9) $$\begin{array}{c} e=\underset{s}{\mathrm{argmin}}\left\{U(x_{m}+d_{c}s,y_{m})\right\}\;\;;s\in \left\{-1,0,1\right\}\\ f=\underset{s}{\mathrm{argmin}}\left\{U(x_{m},y_{m}+d_{c}s)\right\}\;\;;s\in \left\{-1,0,1\right\} \end{array}$$

and $S_{x}=\mathrm{sgn}\left(x-x_{m}\right)$, $S_{y}=\mathrm{sgn}\left(y-y_{m}\right)$ where sgn(.) denotes the sign function. The first part of (8) relates to the case where the cell is free and the second for the case when cell is occupied. If the cell is occupied ($U\left(x_{m},y_{m}\right)=\infty$), then its potential is updated (noted as $p_{cr}^{*}$ in (8)) from already known potential P(x,y) and the gradient g(x,y) in current position ${\left[x,y\right]}^{T}$ as follows:

$$p_{cr}^{*}=P\left(x,y\right)+g\left(x,y\right)\times {\left[x_{m}-x,y_{m}-y\right]}^{T}.$$

Before computing the cell gradients in (8) and (9), a check needs to be made if any of the neighbour cells is outside the environment. If this is the case, the potential of this cell is reconstructed similarly as in (7) considering the known interpolated potential P(x,y) and gradient g(x,y) for the point ${\left[x,y\right]}^{T}$.

From estimated cell gradients $h_{00}$, $h_{01}$, $h_{10}$ and $h_{11}$ (Equation (8)), the final interpolated gradient in current position is obtained by:

(10) $$h\left(x,y\right)=w_{00}h_{00}+w_{01}h_{01}+w_{10}h_{10}+w_{11}h_{11},$$

where the same weights $w_{00}$, $w_{01}$, $w_{10}$, $w_{11}$ as in (3) are used. The comparison of the gradient field g and the improved interpolated gradient h is shown in Figure 3 in the lower graphs. The obtained planned paths by following the interpolated gradient of the potential field result in collision safe and smooth paths as shown in the top-right graph of Figure 3.

Bilinear interpolation can therefore be elegantly used to obtain continuous potential field as well as appropriate desired driving directions (the interpolated negative gradients h) based on a discrete grid-based cost map (discrete CtG potential field). The obtained paths in path planning are collision safe, without local minima and with continuous course of driving direction, which is important for robotic vehicles. Note that Bicubic interpolation [15,36] could also produce smoother interpolations, but it requires 4 by 4 neighbourhood, which is problematic in the vicinity of obstacles or in narrow corridors, as the occupied cells have infinite CtG value. Occupied cells require special treatment before they are used in the interpolation. This becomes even more challenging for bigger neighbourhoods (e.g., 4 by 4 as opposed to 2 by 2). Therefore, for the path planning, we propose the use of bilinear interpolation with appropriate preprocessing of occupied cells and with additional gradient interpolation to obtain smooth paths. The path is smooth, even as it is intuitive and optimal. As mentioned earlier, no local minima will exist in the CtG contour, unlike in the classical APF formulation, because of the inherent manner of its construction. However, if the static map is augmented by new obstacles put in play after its creation, this could change. This eventuality is dealt with in the next section.

Let us analyse the computational complexity of the proposed smooth path planning. The path is obtained with a gradient-descent method. The number of steps that are required to reach the goal is dependent on the size of the sampling step. The sampling step can be lower bounded to prevent oversampling and upper bounded to obtain desired smoothness of the path. Consider that a particular path passes over *M* cells and that the sampling step is selected in a way that on average (or at maximum) *L* iterations of the gradient descent are made in each of the cells. This means that computational complexity of gradient descent is O(LM), and it is therefore dependent on the sampling step and path length. In each step of the gradient-descent calculation, an interpolated value of the gradient is obtained from the four nearest surrounding cells (see Equation (10)) around the current path point. The gradient in each of these four cells is calculated from the APF of the four neighbouring cells (Equation (8)). Therefore, to compute the interpolated gradient for a given cell, a neighbourhood of twelve cells is needed in total. Hence, the gradient does not need to be determined for every cell, but only for the cells that are along the path. We assume that the gradient calculations can be cached; therefore, the computational time and space complexity of obtaining the gradients in the cells around the path are O(M). Note that cell gradient calculations could also be made in parallel if calculation speed is crucial. We calculate the values of the APF for the entire map with *N* cells using Dijkstra algorithm, which in case of a grid map has a computational time complexity of O(Nlog(N)) and final space complexity of O(N). The values of the potential field could also be determined only for the cells in the vicinity of the path that are sufficient for gradient calculation. This is a viable option when we would like to quickly determine only a single path, especially if the map is very large. In this case, the Dijkstra algorithm can be stopped once the goal is reached (in this step, it is also beneficial to use A*) and then the Dijkstra algorithm is resumed only if the value of the potential for an unknown cell needs to be known and only until the final value of the cell potential is known. However, in our case, we calculate the APF for the entire map, as this enables fast recalculation of various paths that lead to a single goal (or begin at a common start), which is beneficial for the cases when part of the map changes, as presented in the next section.

## 4. Discovery of New Obstacles and Reactive Avoidance Manoeuvre

**Figure 4:**
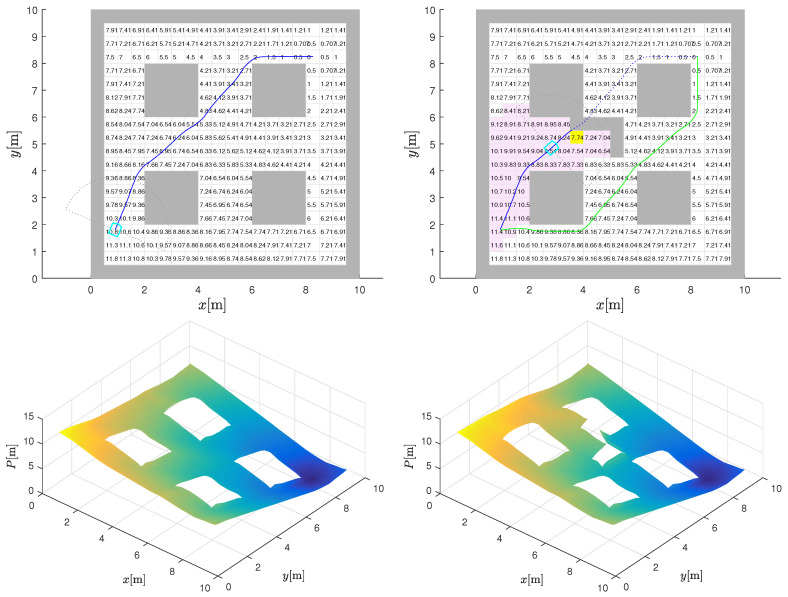
Augmented environment with new discoveries. Static map of environment with planned path in blue line towards goal (**top-left**). Change of environment with new L-shaped obstacle where robot (shown by the sky blue line and its sensor range by the gray dots) is blocked at stuck cell (highlighted by yellow) if continuing based on static CtG map. Replanned CtG values for new environment with highlighted light purple cells where CtG values have changed and new gradient-descent path in green (**top-right**). Interpolated potential (CtG, darker color denote lower values) surface before (**bottom-left**) and after the environment change (**bottom-right**).

A change in the environment is shown in Figure 4 with the addition of an L-shaped obstacle (in Figure 4 (top-right)) cluster roughly halfway through the initially planned path in Figure 4 (top-left). The detection of these additional obstacles is assumed to take place when the robot’s sensor horizon includes the region, through an iterative comparison between what it sees with its sensor and what it expects to see from the initial static map. The range of the sensor used will have some effect on when any reactive manoeuvre is initiated, but this detail is not critically relevant to our model development.

It should be noted that if the new obstacle does not impede motion, then nothing needs to change. However, following the initially planned global path here will stop the robot at the “stuck cell” (see the global path in Figure 4 (top-left) in conjunction with the “stuck cell” shown in yellow in Figure 4 (top-right)), which is effectively a local minimum forced by the new obstacle. Essentially, the robot ends up in a trap in pursuing gradient descent based on the initial plan. In fact, the discovery—here, an addition to the static obstacle map—can be taken as an indication that the CtG values in the vicinity are no longer reliable.

The results of repeating the Dijkstra algorithm to refresh the CtG values of the entire environment with the new obstacles included are also shown in Figure 4 (top-right). The cells with modified CtG values are highlighted (in comparison to plot (top-left)), which clearly reveals that the new obstacles only influence the CtG values (increases them) of a small subset of the cells that are upstream of the new obstacles. The downstream CtG values are unaltered, as would be expected. The new interpolated CtG surface is shown in Figure 4 (bottom-right) and is in accordance with the updated map.

The new gradient-descent path from the original starting point is also shown in Figure 4 (top-right) with the green line, which confirms that if we had been aware of these new obstacles at the very beginning, the global path planning would have accounted for it and the CtG numbers would have been monotonic again. This recalculation could have also been performed from the trapped position of the robot to the goal. However, this is the calculation that is to be avoided because of the associated computational burden. The new path is just presented here to make a point.

**Figure 5:**
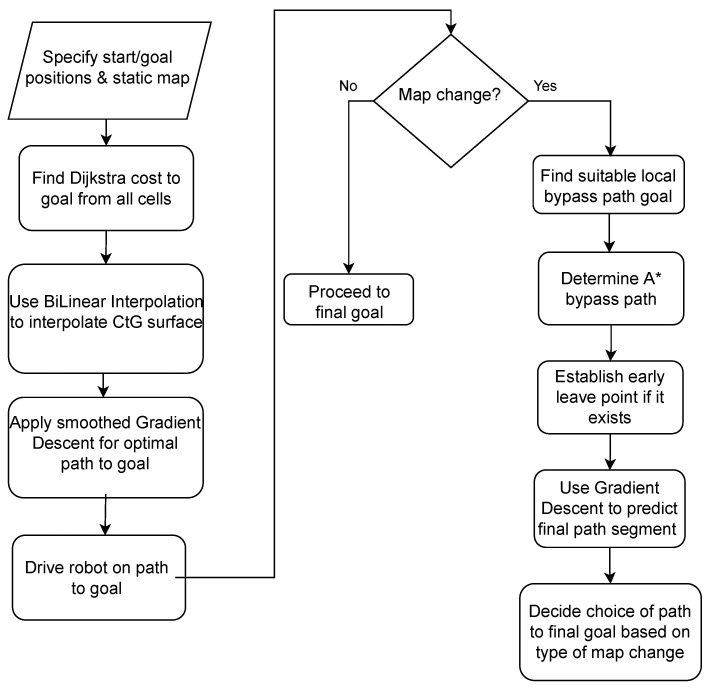
Core flowchart of algorithm (some variations based on case). Leave point determination applies only when current path is blocked.

**Figure 6:**
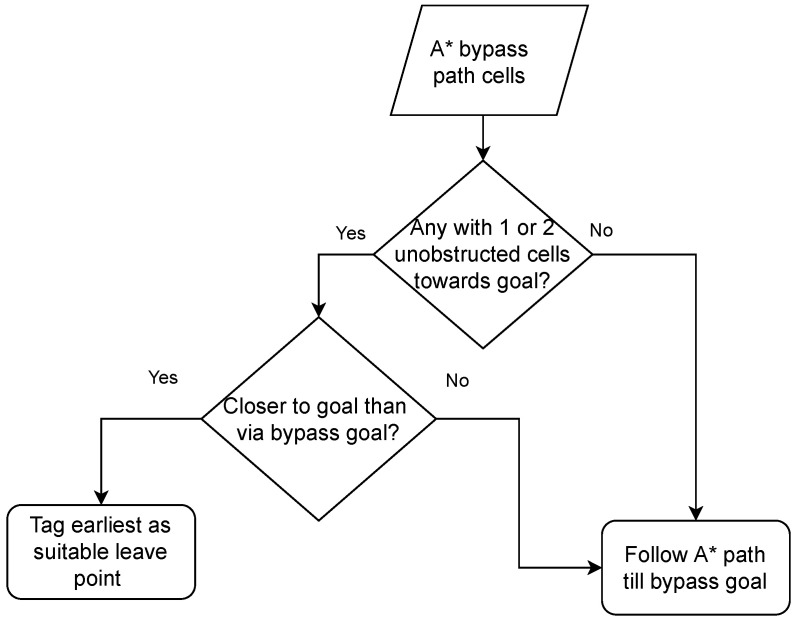
Finding suitable leave point. Leave point is established if cell on A* path has immediate unobstructed cells in original goal direction and is closer to it than path via bypass goal.

**Figure 7:**
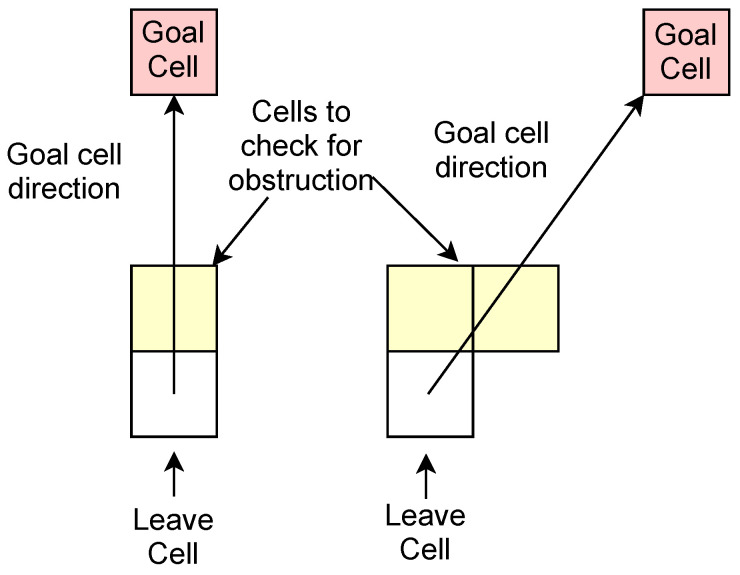
Checking blockage condition for leave cell candidates. Based on alignment of potential leave cell with original goal cell, occupancy status of either one or two cells is checked to determine whether obstruction is present.

Thus, the challenge is to come up with a reactive strategy that enables the robot to get around the obstruction through developing a bypass path that involves minimal computation effort. This path should lead the robot to an area where the old CtG values can be used again to continue travel. The flowchart shown in Figure 5 is a broad representation of the core method adopted. There are minor case-based variations stemming from the type of map change encountered, which are not included in this basic flowchart for simplicity. The explanation that follows is with reference to this flowchart as well as the two in Figure 6 and Figure 7 that follow, dealing with lower-level steps in the algorithm.

**Figure 8:**
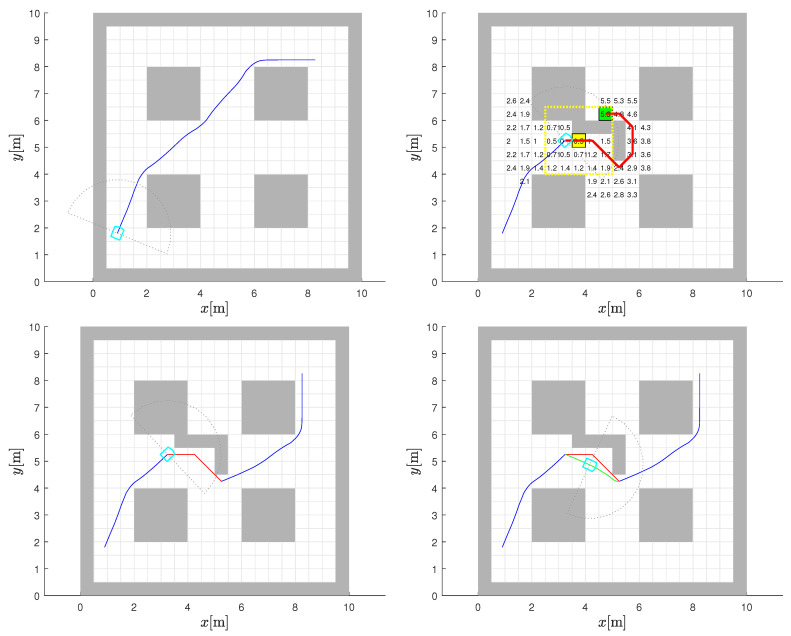
Strategy to negotiate dynamic obstacles. Original planned path based on the static environment map shown is shown with blue line (**top-left**). New obstacle blocking path initiates A${}^{*}$ bypass (red line) calculation from stuck cell (highlighted in yellow) to the temporary intermediate goal cell (highlighted in green) (**top-right**). The leave point and final overall path is shown with A${}^{*}$ bypass (**bottom-left**) and in by smoothed bypass (green line) using BiLI interpolation (**bottom-right**).

A predefined, 5 by 5 search window of cells (see the yellow dashed square in Figure 8 (top-right)), which can be thought of as a “fishing net”, is centred at the stuck robot cell (the yellow cell in Figure 8 (top-right)). The cells within the window are examined to find the lowest CtG value that is smaller than the CtG value at the stuck cell, as per the original static map, to use as a temporary intermediate goal (the green cell in Figure 8 (top-right) to help negotiate the blockage caused by the newly discovered obstacles with a sensor (e.g., LiDAR). An inherent assumption is that the size of the search window is sufficient to uncover a cell with a CtG value that is on the other side of the blockage, and hence unaffected by it. This is facilitated by centring the net at the stuck robot cell right next to the new obstacles blocking the path, even though the blockage may have been detected even further away by the robot’s LiDAR. If this step does not uncover a satisfactory temporary local goal, larger nets are cast iteratively until a suitable cell is found. Moreover, it might be possible to get more creative with the footprint adopted for this search window, which is beyond the scope of this work.

With the stuck cell and the temporary goal identified, the A* algorithm is then run to find a diversionary path from the robot’s stuck location to the temporary local goal identified, as described above. This step should not result in an “unreachable goal” being returned, as the algorithm is executed with the known map at the time. The resulting path obtained is also shown in Figure 8 (bottom-left). It should be noted that using D*Lite instead of A* will not result in any computational savings, because the algorithm needs to be run just once, in which case there is no advantage of one over the other. Whether it is necessary to follow the A* bypass path all the way to completion would depend on the situation. The reason is that if in negotiating the obstacle via the A* path the robot finds itself in a cell that is closer to the final goal, completing the A* path and then proceeding to it is not as efficient compared to treating the cell as a leave point. This additional condition built into the algorithm is laid out in the flowchart extension presented in Figure 6.

A good leave cell must also satisfy another condition. It should also be one for which there is at least one unobstructed cell within its 8-cell neighbourhood in the direction of the goal. This is illustrated in Figure 7 for the two situations that represent all the possibilities that can occur. Essentially there are two cases, because of the quantisation imparted by the discretised cell structure. Either one or two cells need to be checked, depending on the relative positioning of the goal cell and the candidate leave cell. That is, are they vertically or horizontally aligned or at a different angle, in which case the line joining them will be straddled by two cells. In the latter case, only one needs to be free to meet the leave condition. If none of the leave cells pass the dual tests, the bypass path is followed all the way to the temporary bypass goal.

When a leave cell is established, it is taken to mean that the local obstruction has been satisfactorily bypassed and the old CtG values are valid again. The algorithm reverts to the gradient descent using the old CtG values. It should be noted that it is possible to determine a suitable leave point and the final gradient-descent segment even before the robot moves from the stuck cell. This enables the path to be evaluated before travel. The leave point and the final overall path of the robot between the original starting point and goal location are also shown in Figure 8 with A${}^{*}$ bypass (bottom-left) and by the smoothed bypass path using BiLI interpolation (bottom-right).

The use of the CtG values and gradient descent as an overarching method to drive to the goal and the separate handling of unexpected obstructions through a bypass path ensures that, unlike in the classical formulation of the APF, local minima cannot be formed at the global path planning with static map phase. That is, the two-step approach results in local minima being caused only by newly discovered obstacles and transfers the burden of resolving them to the local path-planning phase. This is a key element of the path-planning strategy used here.

The planning model discussed here was evaluated in additional experiments. The environments used and the attendant results obtained are discussed in the next section.

## 5. Additional Experiments and Results

### 5.1. Obstacle Missing from Static Map within Sensor View

**Figure 9:**
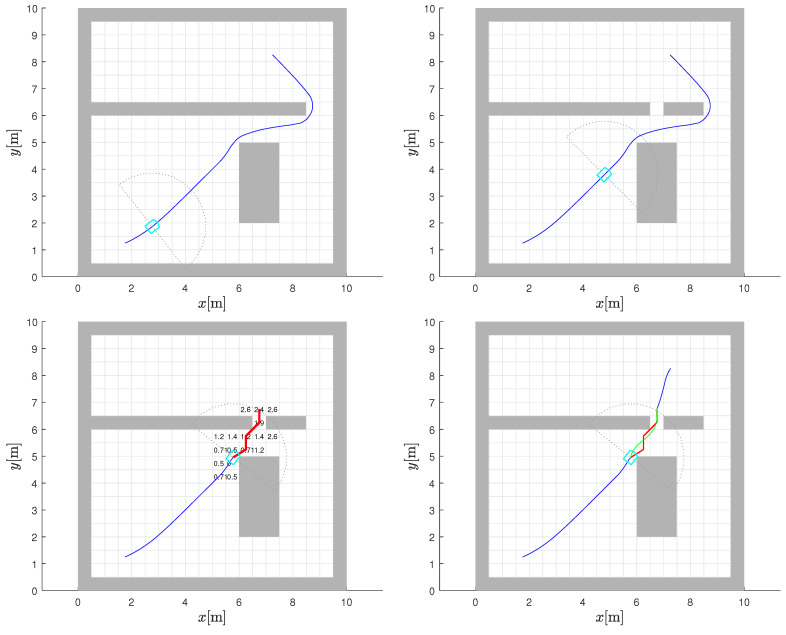
Path planning with obstacle clearance within sensor view. Initial path planned (blue line) based on knowledge of global static map is followed (**top-left** and **top-right**). Robot detects change in static map using sensor view (cleared cell) and computes A* bypass path (red line) to an unblocked cell beyond cleared cell (**bottom-left**). The smoothed bypass path (green line) connects to the gradient-descent path (blue line above the obstacle) based on CtG (**bottom-right**).

A new environment is shown in Figure 9 (top-left) with the start (lower left) and goal (upper right) locations, as well as the path resulting from the global path-planning phase. As the robot travels towards the goal and its sensor horizon advances, it discovers a change in the static map on which the planning was premised. A cell in the “wall” that was thought to be blocked is found to be clear from the robot’s sensor view. Here, the change is a subtraction as compared to the earlier example. The cleared cell as well as the cell where the discovery was made are shown in Figure 9 (bottom-left).

This can be used to trigger an opportunistic strategy—when a blockage is removed, there is a possibility that a shorter path to the goal exists and needs to be investigated. The A* algorithm is invoked to determine a path to the region of the cell, which is now open, so that the old CtG surface can be used again. This is accomplished by setting the target cell for the A* bypass path to an unblocked cell beyond the cleared cell in the direction of the goal. The potential A* bypass path is shown in Figure 9 (bottom-left) in red and the corresponding smoothed version by the green line in Figure 9 (bottom-right). The remaining path from that cell to the original goal cell is again obtained through using the CtG values and gradient descent and is shown in Figure 9 (bottom-right) in which the overall path from the original starting position to the goal is also evident.

If there are multiple occupied cells that are now clear, all of them need to be assessed in the same manner as discussed above. Before taking a bypass path, a check needs to be conducted to see whether the modified path (made up of an A* segment followed by a gradient-descent segment) is shorter than the remaining current path to the goal. Only if the newer path is better should the robot use it to get to the goal instead of the original path. In the environment considered, this happens to be the case.

### 5.2. Obstacle Missing from Static Map Outside of Sensor View

It is also possible to conceptualise a situation where the robot comes to know about the removal of an obstacle from the static map in a region of the environment outside the sensor range of the robot while it is in motion on the current path to the goal. For example, this could happen when another robot passing by that region notices and relays the change(s) to a central station and/or all agents. While this could be a corrected error in the map creation, it could also be the result of a temporary obstacle (for example, a fallen tree) being cleared.

This situation can be handled in the same way as the previous one. A potential A* bypass path can be estimated from the robot’s current position to a target cell just beyond the cell whose occupancy status has changed. This serves as a bridge to an area where the old CtG values are still valid. As before, the next segment of the path is established using the gradient descent from the temporary bypass goal on to the original destination. Then, based on whether this new alternate route is shorter than the remaining part of the current route, the robot can decide whether to switch to the alternate path or continue on the current one.

**Figure 10:**
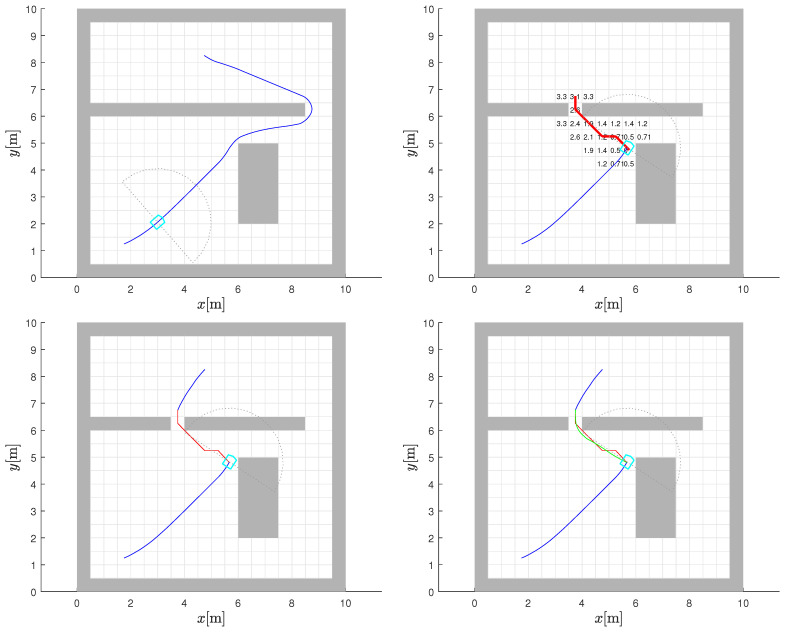
Path planning with obstacle cleared outside the robot sensor view. Initial path (blue line) planned based on knowledge of global static map is followed (**top-left**). Robot is informed of cleared obstacle cells (outside robot’s sensor view) and A* bypass path (red line) to an unblocked cell beyond cleared cell is computed (**top-right**). The composite alternative path made up of final gradient-descent path (blue line above the obstacle) using the original CtG (**bottom-left**) and smoothed bypass version (green line) (**bottom-right**) is shorter than the one in the (**top-left**) figure and robot switches to it.

An example of this case is shown in the environment in Figure 10. The various parts of the figure are in line with Figure 9 and can be understood with the help of the caption. In the environment of Figure 10, the information on the map change helps chart a shorter route to the goal.

### 5.3. U-Shaped Trap

**Figure 11:**
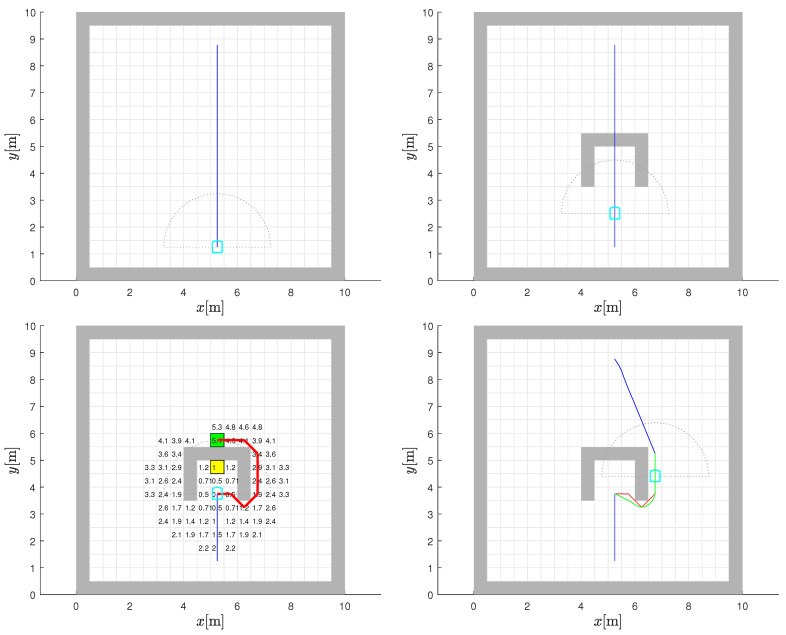
Path planning with appearance of unexpected U-shaped trap. Robot initially plans path (blue line) in empty static map (**top-left**) and starts to travel on it (**top-right**). When blockage is within sensor range, a bypass path is computed by a red line (**bottom-left**). The path is smoothed (green line) and connects with the final gradient-descent path (blue line) using CtG (**bottom-right**).

The last environment considered is one which incorporates the classic U-shaped concave trap. The static map is initially empty and the planned path between the start position and the goal is shown in Figure 11 (top-left), before the blockage is discovered, and is as expected. Even when the lower part of the U-shaped particle is discovered (Figure 11 (top-right)), the robot continues to proceed on the initial straight path recommended by global path planning. It does this until it encounters the core structure of the trap and the attendant local minima created (Figure 11 (bottom-left)). The ability to resolve an unexpected concave obstacle configuration on the planned path is a good test of the ability of an algorithm, because those that are purely combinatorial will fail this test.

In accordance with the algorithm, a 5 by 5 window of cells is examined around the stuck cell (the yellow cell in Figure 11 (bottom-left) where the robot’s initial gradient-descent path is blocked by the obstacle) to find the lowest CtG value below the current one (temporary intermediate goal cell marked by green in Figure 11 (bottom-left)). As explained earlier, such a cell is considered as being in an area where the CtG values are unaffected by the new blockage. If in some other case, the chosen 5 by 5 search window size does not produce a suitable target, because the cell with a minimum CtG value smaller than the current value lies within the trap zone, the search can be repeated using a larger 7 by 7 window and so on. This will eventually yield a temporary bypass goal outside the trap. The A* bypass path to this cell is also shown in Figure 11 (bottom-left) in red. The smoothed version obtained by the bilinear gradient interpolation in green, the post-bypass path segment based on the gradient descent, and the overall composite path between the start and goal points is shown in Figure 11 (bottom-right). Note that the path follows the computed bypass only until the leave point where the old CtG values and its interpolated gradient-descent path can be followed again.

## 6. Discussion

Although bilinear interpolation and the calculation of gradients from a discrete grid are well-established in image processing, their direct application to a discrete APF can lead to several problems. At the points where the cell is connected to its neighbour, discontinuities can occur in the gradients, making the use of a gradient descent problematic. This can lead to undesirable zigzag paths when following the direction of the gradient descent. This problem is much more pronounced near obstacles. The potential of occupied cells is not defined by the CtG assignment and can be considered infinite, as the cell should not be part of a path to the goal. This can lead to problems with local minima near obstacles where the direction of the gradient descent could change by more than 90°.

To interpolate the potential appropriately, one could also use some other higher-order interpolation technique, such as the bicubic interpolation [15,36]. The advantage of this technique would be a smooth gradient transition at the cell border because it uses third-order polynomials for the interpolation, which are continuous up to the second derivative (C${}^{2}$). However, bicubic interpolation requires the use of a 4 by 4 neighbourhood, which becomes problematic near obstacles or in narrow corridors because the occupied cells have infinite (undefined) potential values. These occupied cells need special treatment before they can be used in the interpolation. Therefore, bicubic interpolation brings its own problems, as it requires a neighbourhood of 16 cells for the interpolation, in contrast to bilinear interpolation, which only requires 4 cells.

An additional problem that plagues bicubic interpolation is the occurrence of anomalies such as surface oscillations and the possibility of local minima near obstacles. Third-order polynomials have a continuous gradient which, while fitting the equidistant cell centres near obstacles (e.g., obstacle corners), causes oscillations in between (a common problem in the interpolation where the fit is perfect at the data point but could be oscillatory in between, known as the Runge phenomenon).

In image processing, the gradient is normally computed with the convolution of the image with the gradient operator. There are various gradient operators, such as one-dimensional operators (e.g., [1,−1], [1,0−1]) and Robert’s cross or the Prewitt, Sobel and Scharr operators [37], which are more robust to noise. Some of these filters introduce gradient shifting and/or smoothing. In our case, averaging is not required, as the APF inherently does not contain noise. The proposed method for calculating the APF gradient is therefore different from the gradient methods used in image processing because it is designed in a way that the gradient in the cell centres always points towards the neighbouring cell with the lowest potential, or the gradient is zero if the current cell has the lowest potential. This ensures that the interpolated gradient points towards the cells with the lowest potential, regardless of the potential magnitude in the cell neighbourhood (without an undesired gradient shift and averaging). Therefore, an optimum smooth path from the start to the goal can be obtained, because the obtained path accurately follows the bottom of the valley that is defined by the APF.

The calculation of the potential field and the gradient in the free space away from the obstacles is straightforward, but in the vicinity of the obstacles, special care is needed to determine the appropriate potential and gradient, as presented in the paper. In the cells surrounding the obstacles, the gradient is calculated from the potential field of the neighbouring cells. If some of these adjacent cells are occupied by obstacles, the potential in these cells may be different depending on which side of the obstacle the gradient is calculated—this occurs in the case of thin or diagonally touching obstacles. Therefore, the batch calculation of the APF can only be performed for the cells that do not touch the obstacles. Moreover, to determine the optimal path, the proposed approach can calculate the gradients online only for the cells that are surrounding the tip of the path while it is being generated. One could resample the grid to double/quadruple the resolution of the map to alleviate the problems encountered near thin obstacles. However, this would require more computational resources (by a factor of four in the case of a double resolution) to calculate the APF and its gradient. However, because the gradient is interpolated, smooth paths are obtained even if the resolution of the map is low.

The proposed bilinear interpolation is applied to the path planning in a static and dynamic environment. A simple model for combining the global and local path planning that also derives from the original potential fields concept is used. Its key aspects are as follows. A method is needed for multiple missions that could potentially require navigation to the same destination in the environment. A static APF is therefore interpolated based on the pre-calculated CtG values for the cells navigating the path to the goal. A global plan is created based on these CtG values using a gradient descent on the static APF. Along its path, local map changes in the environment can be detected in various ways. A bypass strategy is formulated that enables the robot to find and evaluate a temporary bypass path through the use of the A* algorithm. A case-based decision is made whether to take the alternate path. Obtaining the diversionary path is relatively fast compared to regenerating CtG values for the entire environment. The benefits will be proportionately even greater for larger and less constricted environments.

In summary, the proposed approach introduces the following contributions/modifications in APF-based path planning: a small required neighbourhood (2 × 2, compared to other interpolations), an easier treatment of the occupied cells in the interpolation, and no anomalies that could result in local minima or the oscillating direction of the gradient-descent path near obstacles. The basic bilinear interpolation has a discontinuous gradient between cells, which we take into account by our proposed additional interpolation for gradients. Standard image processing techniques usually apply a batch calculation to the entire image, which simplifies the algorithmic flow in an obstacle-free area. However, additional special care is required to determine the appropriate potential and gradient near the obstacles. The proposed approach reduces the computational effort as the interpolation is only performed on cells along the path and not for the entire environment as is usually the case with batch image processing algorithms.

Due to the applied interpolation in the potential function, good quality paths are obtained even at a rough grid resolution of the environment. These contribute to the computational and memory requirement efficiency of the approach.

## 7. Conclusions

This work proposes a new approach to construct a navigation function in a variant of artificial potential fields (APF) that can be applied to navigation, path planning and the control of a mobile robot. The navigation function guarantees safe guidance to a goal without local minima in concave traps, which is a common problem with APFs. Optimality and convergence are inherited from an optimal grid-based search which results in a discrete APF.

To obtain a smooth navigation function and the associated gradient-descent driving direction, we apply a bilinear interpolation with several novel extensions that allow an efficient application to path planning. First, we propose a reconstruction of the discrete potential for the neighbourhood of cells used in the interpolation that belong to obstacles or are outside the environment. Second, we introduce an additional interpolation of the gradient-descent directions from the estimated discrete gradients of the interpolated cells. This leads to a smooth, optimal and collision-safe path or navigation towards the goal. Third, the proposed interpolation approach can be performed online and is computationally efficient as it only interpolates the discrete cell potentials along the planned path.

Several path planning results are provided to illustrate the performance of the navigation function. To illustrate the application in dynamic environments, we propose a simple strategy to combine global and local path planning to bypass detected dynamic changes. The strategy enables a robot to find and evaluate a temporary bypass path around newly detected obstacles through the use of the A* algorithm.

## Data Availability

Not applicable.
